# Comparing the Speech Perception of Cochlear Implant Users with Three Different Finnish Speech Intelligibility Tests in Noise

**DOI:** 10.3390/jcm10163666

**Published:** 2021-08-19

**Authors:** Tytti Willberg, Ville Sivonen, Pia Linder, Aarno Dietz

**Affiliations:** 1 Department of Otorhinolaryngology, Turku University Hospital, 20521 Turku, Finland; 2 Institute of Clinical Medicine, University of Eastern Finland, 70211 Kuopio, Finland; 3 Department of Otorhinolaryngology—Head and Neck Surgery, Head and Neck Center, Helsinki University Hospital and University of Helsinki, 00029 Helsinki, Finland; ville.sivonen@hus.fi; 4 Department of Otorhinolaryngology, Kuopio University Hospital, 70029 Kuopio, Finland; pia.linder@kuh.fi (P.L.); aarno.dietz@kuh.fi (A.D.)

**Keywords:** speech perception, speech perception in noise, cochlear implant, matrix sentence test, digit triplet test, outcomes, adult, hearing loss, audiology

## Abstract

Background: A large number of different speech-in-noise (SIN) tests are available for testing cochlear implant (CI) recipients, but few studies have compared the different tests in the same patient population to assess how well their results correlate. Methods: A clinically representative group of 80 CI users conducted the Finnish versions of the matrix sentence test, the simplified matrix sentence test, and the digit triplet test. The results were analyzed for correlations between the different tests and for differences among the participants, including age and device modality. Results: Strong and statistically significant correlations were observed between all of the tests. No floor or ceiling effects were observed with any of the tests when using the adaptive test procedure. Age or the length of device use showed no correlation to SIN perception, but bilateral CI users showed slightly better results in comparison to unilateral or bimodal users. Conclusions: Three SIN tests that differ in length and complexity of the test material provided comparable results in a diverse CI user group.

## 1. Introduction

Cochlear implantation (CI) has been a standard treatment for severe-to-profound hearing loss (HL) for more than two decades. CIs are safe and effective intervention for HL [1,2,3,4], even in elderly patients [5]. Reaching open-set speech perception is currently a realistic and attainable goal for the majority of CI users [6,7,8]. However, while CIs provide most patients adequate speech perception in quiet listening conditions, speech perception typically deteriorates drastically in noise [6,9], and noisy listening environments remain a challenge for CI users.

Testing CI users’ speech perception both in quiet and in noise is essential for obtaining a comprehensive view of hearing in everyday listening conditions. Many current national and international guidelines recommend including some type of a speech-in-noise (SIN) test in the test batteries used for evaluating CI candidacy and rehabilitation outcomes [10,11,12]. However, whereas audiometry is a standardized procedure that provides reliable and comparable results across languages and diverse patient populations, SIN tests are language specific and subject to multiple confounding factors, which complicates the interpretation of their results [13].

A plethora of different types of SIN tests are available for many languages to accommodate diverse patient and testing needs [14,15,16,17]. For example, complex sentences better reflect everyday conversations than isolated words, and sentence-level tests are therefore usually preferred when testing in the clinic. On the other hand, children and adults with limited language knowledge benefit from shorter speech materials with familiar words [18,19]. The COVID-19 situation has increased the need and interest for tele-audiology and remote hearing testing [20,21]. Luckily, simple, closed-set test materials (i.e., digit strings) are well suited for remote testing [15,22], and remote testing is feasible and reliable with current CI technology [23,24].

One of the confounding factors for interpreting SIN test results is the background noise that can vary from a steady state [17,25,26] to a fluctuating noise or emerge as speech babble [16,27]. Speaker selection also introduces variability, as some tests only use one speaker with clear articulation [25,26,28], while others have multiple speakers using different dialects [27]. These differences in SIN test content and construction significantly affect the test scores. Therefore, SIN test scores that are typically expressed either as a percentage of correctly recognized words or as speech reception threshold (SRT) are test-specific.

Only a few studies have compared two or more different sentence-level SIN tests in the same hearing-impaired patient population [29,30,31]. Data from a study by Dillon et al. [31] demonstrate how differences between test-specific characteristics lead to different results in the same patient population. When a group of CI users were tested with the Hearing in Noise Test (HINT) [25], a SIN test that uses simple, short everyday sentences presented in a steady background noise, the speech recognition scores in noise were, on average, between 80–100%. However, when the same CI users conducted the AzBio sentence test [16], which uses more complex sentence material presented in babble-noise, the scores were 20–60%.

Even the same SIN test can produce different results if different test protocols are used. For example, at +10 dB signal-to-noise ratio (SNR) a group of CI users reached a mean score of 46.8% for the AzBio sentence test, while at a more difficult SNR of +5 dB, the mean result was only 23.3% [32]. Some SIN tests have a strictly defined, standardized protocol that clearly states which SNRs to use [17,25]. Clinical guidelines also provide recommendations for a standardized use of the tests [10]. However, a recent survey revealed that the recommended procedures are not always followed in the clinic [33], which can lead to biased results and further complicate their interpretation.

Comparing CI users’ speech perception results from two different SIN tests is difficult without intricate knowledge of both tests, testing conditions, and the typical CI user performance range for both tests. Often, when a new SIN test is developed, the only reported reference value is the normative reference value for normal-hearing (NH) listeners [17,25,27]. However, NH listeners typically reach 100% speech intelligibility even with the more complex SIN tests [34]. Since restoring normal speech perception in noise is not possible with the current CI systems, CI users usually fail to reach the normative range for most SIN tests [2,32,35,36]. In addition to reference data for NH listeners, reference data for CI users would be valuable in the clinical practice. The variability in CI rehabilitation results is considerable [1,3,37], and the concept of CI user reference data is complicated, as it is inevitable that the average performance of a large CI patient population may be an unrealistic goal for some CI users and an underachievement for others. Nevertheless, the need for CI user reference data that is collected from a large, unselected patient population under standardized, well-defined testing conditions is recognized in multiple recent publications [12,33,38].

Ideally, all speech perception in noise assessments could be carried out with one, internationally comparable SIN test. However, due to differences in patient-related factors (i.e., age, memory span, language knowledge), test environments (clinic vs. home), and testing requirements (accurate clinical diagnostic vs screening), no single test can currently accommodate all of these needs. To meet the requirements of a comprehensive SIN test battery, three SIN tests have been developed in Finnish. The Finnish matrix sentence test (FMST) [26] is the standard for SIN testing in Finnish, with reference data for CI users published in 2015 [39]. The two more recently developed SIN tests, the Finnish simplified matrix sentence test (FINSIMAT) [40] and the Finnish digit triplet test (FDTT) [41] have not yet been validated for CI users. Our first aim was to validate these tests in a clinically representative CI patient population by assessing their correlation with the FMST. The second aim of our study was to provide reference data for the new tests in CI users.

## 2. Materials and Methods

### 2.1. Participants

The participants in this study were 80 consecutive adult and adolescent CI users from Kuopio University Central Hospital and Helsinki University Central Hospital, who volunteered to participate in the study during their routine follow-up visit. Data were collected between December 2016 and February 2019. Altogether 83 CI users met the inclusion criteria (native Finnish speakers, no cognitive impairments, at least four months of CI experience). However, three CI users had to be excluded since their speech recognition score for the FMST presented at +10 dB SNR was less than 70%. The reason for exclusion was the adaptive test procedure the FMST uses; the adaptive test procedure converges to a SNR where 50% of the test items are correctly recognized, and it only functions reliably if the majority of the test items are recognized at +10 dB SNR [7]. The study was approved by the Research Ethics Committees of the Hospital District of Northern Savo and HUS, and informed consent was obtained from all of the participants.

### 2.2. Speech Intelligibility Tests

The differences between the three tests are summarized in Table 1. The FMST [26] is based on the Oldenburg Sentence Test (OLSA), and the general principles of this type of matrix test are described in detail elsewhere [17,42,43,44]. The test material consists of 50 common everyday words combined into five-word sentences. All of the sentences have the same structure, but the content is semantically unpredictable (e.g., “Johanna buys seven large cups.” or “Harri sees four old cars.”). The FINSIMAT [40] is based on the simplified version of the OLSA, the Oldenburg Kinder Satztest [45]. Simplified matrix sentence tests use a shortened version of the original word matrix to form three-word pseudo-sentences (i.e., “seven large cups” or “four old cars”). The third SIN test in our study, the FDTT [41], uses digit triplets (i.e., 2-7-1) as speech material. It is based on a test originally developed by Smits et al. [15] for large-scale hearing screening. 

All of the tests use speech-shaped background noise that is created by superimposing the test material 30 times. All of the tests can be conducted at a fixed SNR, but the preferred clinical practice is to use an adaptive test procedure, where the presentation level of the next speech item is based on the responses for the previous items. The FDTT uses a fixed step size of 2 dB, and the whole triplet needs to be recognized for the answer to be considered correct. The FMST and the FINSIMAT use word scoring, and the step size changes based on how many of the words in the (pseudo)sentence were correctly recognized [46]. When using the adaptive test procedure, the results are expressed as a SRT estimate, which is the SNR that is needed to correctly recognize 50% of the test material. For the FMST and the FINSIMAT, the test software obtains the SRT estimates by fitting the test data to the test-specific psychometric function. For the FDTT the SRT estimates are calculated by averaging the SNRs of the last 27 triplets. In our clinical procedure, the noise is held constant at 65 dB SPL, and the starting SNR is 0 dB for all of the tests.

### 2.3. Test Procedure

The participants conducted all of the tests during a single visit with their preferred device configuration (CI, CI + HA or CI + CI) and instructions to use their preferred device settings. All of the measurements were conducted in a sound field with the participants seated at 1 m distance in front of the loudspeaker that was used to present the signal and the noise (S0N0). Testing began with two practice measurements with the FMST: the first FMST test list was presented at +10 dB SNR, and the second test list using the adaptive test procedure. The practice phase was followed by the actual test measurements: one test list was conducted for each test using the adaptive test procedure. The test material was presented in a counterbalanced order across participants. The participants received no feedback during the testing, but they were told in advance which test would be conducted next. Before the practice phase and the test measurements with the FMST and the FINSIMAT, the participants were shown the word matrix in writing for each test in order to (re)familiarize them with the words, but the written word matrices were not available during the actual test measurements.

### 2.4. Statistical Analysis

The baseline characteristics and the final results were described with standard descriptive statistics. Frequency and relative frequency were used to describe categorical variables. Normal distribution was tested using the Shapiro-Wilk test. When the data were normally distributed, means, standard deviations (SD), and ranges were used describe the continuous variables. Medians and ranges were used for variables with non-normal distribution.

Comparisons between the different device configurations were performed using one-way analysis of variance (ANOVA) with Sidak correction for multiple comparisons for post-hoc tests. Based on the normality of the results, the Pearson correlation coefficient (r), or Spearman’s rank-order correlation coefficient (r_S_) was used to assess the strength of association among the continuous variables. Data were analyzed for all of the participants together and, for additional analysis, the participants were divided in to two age groups: (1) age ≤ 65 and (2) age > 65 years. IBM SPSS Statistics (Version 27) was used for all statistical analysis with a significance criterion of *p* ≤ 0.05.

## 3. Results

The demographics of the participants are summarized in Table 2. The mean age of the participants was 56 years (SD = 16.7). Of all the participants, 55 (69%) were 65 years of age or younger. Detailed hearing history was not recorded as our aim was not to assess explanatory factors behind the results, but to compare the tests to each other. The range for CI experience was wide, as the most recent CI recipient had only five months of CI experience while the most experienced user had used a CI for more than 18 years. Med-El was the most commonly used CI manufacturer (49% of all participants). All of the participants were fitted with current CI and HA technology at the time of the data collection. The CI processors used were Naída CI Q70/90 (Advanced Bionics); Opus 2, Sonnet, Rondo (Med-El); CP810, CP910, Kanso (Cochlear). All other data were normally distributed except for CI experience, recognition scores at +10dB SNR, and age in the two age groups.

For the FMST, the SRT estimates showed a large variance in the participants’ speech perception, with results ranging from +0.8 to −7.7 dB SNR. A similar variance was observed for the FINSIMAT (+0.1 to −9.1 dB SNR) and the FDTT (−0.4 to −8.2 dB SNR). The mean SRT estimates for the tests are summarized in Table 2.

**Figure 1:**
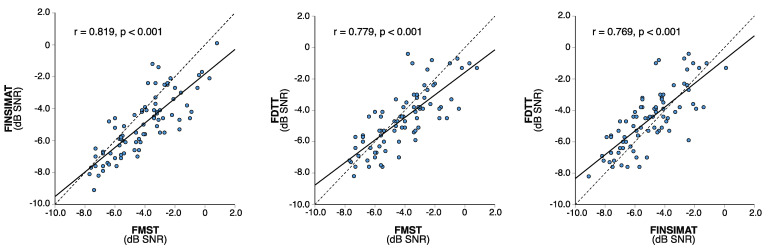
Speech reception threshold (SRT) estimates in dB signal-to-noise ratio (SNR) plotted against each other for the three-speech intelligibility in noise tests with a linear regression plotted as a solid line. Pearson correlation coefficients (r) and *p*-values are included for all regression plots. FMST, Finnish matrix sentence test; FINSIMAT, Finnish simplified matrix sentence test; FDTT, Finnish digit triplet test.

**Figure 2:**
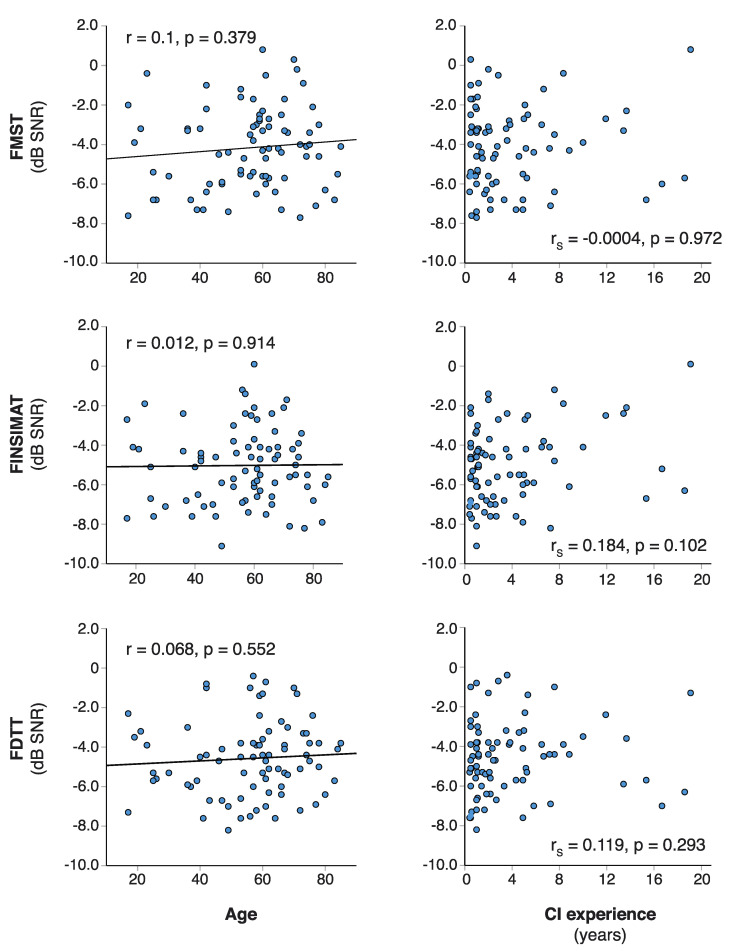
Age and cochlear implant (CI) experience plotted against the speech reception threshold (SRT) estimates for all three tests. For correlations to age, a linear regression is plotted as a solid line, and Pearson correlation coefficients (r) with corresponding *p*-values are included. Spearman’s rank-order correlation coefficient (r_S_) was used for CI experience data.

**Figure 3:**
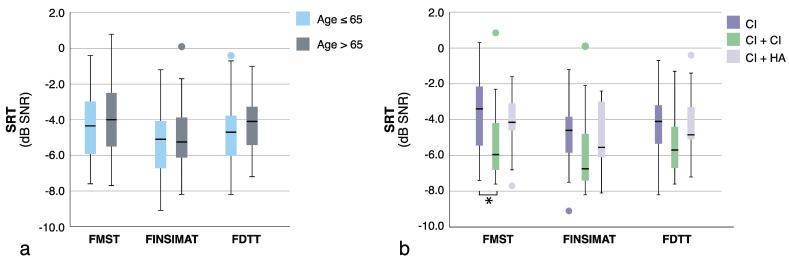
Comparisons between the different age groups (**a**), and device modalities (**b**). The asterisk notes the only statistically significant difference between the results. FMST, Finnish matrix sentence test, FINSIMAT, Finnish simplified matrix sentence test; FDTT, Finnish digit triplet test; CI, cochlear implant; HA, hearing aid.

Figure 1 shows all the SRT estimates plotted against each other. All of the test scores were strongly correlated, and the correlations were statistically significant (*p* < 0.001 for all). The strongest correlation was between the FMST and the FINSIMAT (r = 0.819), and it was only slightly weaker between the FMST and the FDTT (r = 0.779). The FINSIMAT and the FDTT had an equally good correlation (r = 0.769). No correlation was observed between the SRT estimates and age, or between the SRT estimates and the CI experience (Figure 2). The differences in the SRT estimates between the younger and older CI users were small (Figure 3a), and statistically insignificant for any of the tests (t_FMST_ (78) = −0.503, *p* = 0.617; t_FINSIMAT_ (78) = 0.384, *p* = 0.702; t_FDTT_ (78) = −0.679, *p* = 0.499).

**Figure 4:**
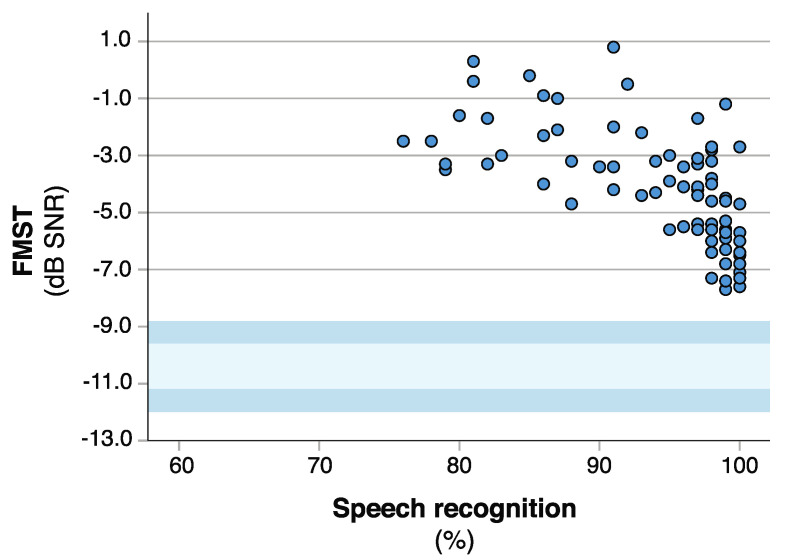
Speech recognition scores at +10 dB plotted against the speech recognition threshold estimates obtained with the adaptive test procedure for the Finnish matrix sentence test (FMST). The light blue represents the normative range ±1 standard deviation (SD) for the FMST, and the dark blue areas denote the ±2 SD range.

Participants with bilateral CIs had better SRT estimates for all of the tests (Figure 3b). However, the differences were only statistically significant for the FMST (F(277) = 6.311, *p* = 0.003), and the post-hoc comparisons only showed a statistically significant difference between unilateral and bilateral CI users (*p* = 0.002). A ceiling effect was observed for the FMST presented at +10 dB SNR, but not when using the adaptive test procedure (Figure 4).

## 4. Discussion

Our aims were to validate the FINSIMAT and the FDTT in CI users by comparing the results to the FMST, and to collect reference data for the two tests in an unselected and representative CI patient population. Despite differences in speech material and difficulty level, we observed concise and correlating results in our heterogenous patient population for all three of the Finnish SIN tests.

The mean SRT estimates for all three tests in our CI patient population showed a similar pattern to the normative reference values (see Table 1). For NH listeners, the reference value for the FMST is the highest, whereas the reference value for FINSIMAT is the lowest (i.e., most negative), and the reference value for the FDTT is situated in-between. Figure 3b demonstrates the differences between the three SIN tests by showing how the mean SRT estimates for the FMST were always the highest, and the mean SRT estimates for the FINSIMAT were the lowest, despite the differences in the overall performance level between unilateral, bilateral, and bimodal CI users.

The differences in the SRT estimates between the three tests are likely due to the differences in speech materials and test procedures, since all of the tests used the same female speaker. The FMST is the most complex of these tests due to longer sentences and relatively long test-lists (20 sentences per test-list). The test is precise and suited for an accurate diagnostic assessment in patients with hearing loss, since the test-retest value was within 1 dB for CI users [7,39]. The results we obtained with the FMST are similar to previously published results for CI users. In the validation study for the FMST [39], the mean SRT estimate for the participants was −3.5 dB SNR (SD = 1.7). Our participants reached a slightly better mean SRT estimate of −4.2 dB SNR (SD = 2.1). However, during the 2015 validation study [39], all of the participants were tested in the unilateral CI condition, whereas in the present study all of the participants were measured with their preferred device configuration, and thus only 55% of our participants were measured in the unilateral CI condition. The mean SRT estimate for the FMST for our unilateral users (−3.6 dB SNR (SD = 1.9)) did not differ from the original validation data. Sivonen et al. [47] reported a slightly better mean SRT estimate of −4.6 dB SNR for a selected group of unilateral CI users. For a group of CI users with good functional low-frequency hearing preoperatively, Iso-Mustajärvi et al. [48] reported a mean post-operative SRT estimate of −5.2 dB SNR for the FMST, reflecting the significance of residual hearing and hearing preservation surgery in the CI performance.

The FINSIMAT has the lowest SRT estimate, both in NH listeners as well as in our patient population (see Table 1 and Table 2). The speech material for the FINSIMAT is simple and restricted. The short pseudo-sentences are easier to retain in memory during the listening task than the longer FMST sentences. These qualities likely contribute to the lower reference value for NH listeners, and also to the lower SRT estimate in our study population. The shortness and simplicity of the test is essential for testing children, since their speech perception in noise is still maturing [19,49], and children with hearing loss may have a shorter auditory memory span [50,51]. Likewise, these qualities might render the test the most reliable SIN measure in the elderly, since ageing and age-related cognitive decline affect the speech perception in noise independently of the hearing level [52,53,54]. In the clinical protocol for the FMST, two practice lists are conducted before the actual test measurements, and sometimes after the practice, some elderly patients are too tired to focus on the listening task during the actual test measurements, which may affect the reliability of the results. Therefore, we are currently assessing whether the shorter FINSIMAT would provide more reliable results in this patient population [40].

Plenty of data have been published on the use of the standard matrix sentence tests in CI users [48,55,56,57], but the present study is, to our knowledge, the first on the simplified matrix sentence tests in adult CI users. The results for CI users are similar to the results from hearing impaired adults, who also showed slightly better SRT estimates for the FINSIMAT than for the FMST [40]. In that study, the FINSIMAT was presented monoaurally with headphones, and the mean SRT estimate for the unilateral test condition was −4.1 dB SNR (SD = 2.0). The result is slightly worse than the mean SRT estimate for our participants (−5.0 dB SNR (SD = 1.9)).

The FDTT was originally developed for hearing screening [15], but it has been increasingly adopted into hearing diagnostics in recent years [23,24]. The DTTs are especially well suited for remote testing since responses can easily be recorded using a picture of a number pad on a touch-screen device. Even though the speech material for DTTs is extremely simple and restricted, previous studies have reported good correlation between DTTs and other SIN tests in CI users [23,58]. To our knowledge, ours are the first published data to compare a DTT to a matrix sentence test in CI users.

Even though all of our tests were designed for different purposes and patient populations, and despite producing SRT estimates that are not directly comparable, they were all designed to measure the same thing: the ability to perceive speech in noise. All of the test results should therefore correlate with each other, and we did observe strong and statistically significant correlations between all of the tests. Since a previous validation study has already confirmed that the FMST is well-suited to assessing speech perception in CI users [39], the FMST was used as a reference point for data from the other tests. For the FINSIMAT, the correlation with the FMST was 0.82 (*p* < 0.001), which is similar to previously published results from elderly listeners with mild-to-moderate hearing impairment (r = 0.89, *p* < 0.001) [40]. For the FDTT, the correlation with the FMST was equally good (r = 0.78, *p* < 0.001), even though the speech materials differ significantly in length and complexity. A previous study reported similar correlation (r = 0.81) between the French DTT and the French matrix sentence test in hearing-impaired adults [30].

Since all of our participants had at least four months of CI experience, it was not surprising that CI experience did not correlate with the SIN perception. What was striking, however, was the lack of correlation between the patients’ age and SIN perception (see Figure 2). The current policy in Finland is to provide two CI for patients under the age of 65 if the benefit from bimodal fitting is insufficient. With some exceptions (i.e., extremely poor vision), patients over the age of 65 typically only receive one CI. This is reflected in our data, as the proportion of bilateral CI users in participants under the age 65 (33%) was twice of that in older participants (16%). Despite this, the differences in the SRT estimates between the two age-groups were small and statistically insignificant. Multiple studies have reported excellent outcomes in elderly CI users [5,59], and our data corroborate these findings.

Ceiling effects are a well-known problem with speech perception tests in quiet and SIN tests that used fixed presentation levels [6], and we also observed a ceiling effect for the FMST presented at +10 dB SNR. For tests that use a fixed presentation level, a more difficult SNR can be used to prevent ceiling effects, but, on the other hand, this predisposes patients with poor speech perception to floor effects [32]. Adaptive test procedures can more easily accommodate a large performance range, as our data demonstrates. None of our participants reached the reference range for NH listeners (Figure 4), and only three participants had to be excluded for having inadequate speech perception to conduct the SIN tests with the adaptive test procedure. With the adaptive test procedure, the FMST was also more accurate in detecting individual differences between the participants; Figure 4 demonstrates how, despite reaching perfect or near perfect speech perception at +10 dB SNR, the speech perception significantly deteriorated at more adverse SNRs, leaving some patients performing very poorly (SRT of −1.2 dB SNR), while others reached nearly the performance range of NH listeners (SRT of −7.7 dB SNR).

## 5. Limitations

Studies on SIN tests often include a speech intelligibility test in quiet with short words (CNC words, for example) for comparison. We were not able to include any such speech intelligibility measures, since the only available word-level speech intelligibility test in Finnish is outdated, and the test lists differ in intelligibility [39]. Additionally, since the FMST is the only sentence-level SIN test in Finnish, we were not able to include a SIN test with everyday sentences for comparison. An interesting addition to the current data set would have been a subjective evaluation of hearing and hearing related quality of life to assess how well the test results correlate with CI users’ subjective evaluation of their functional hearing.

Since the main aim of our study was not to compare different device modalities, but to assess how well the three SIN test results correlate in different test conditions, we allowed our participants to use their preferred device configuration without assessing objectively, whether it was their best aided condition. All of our bimodal users used their HAs regularly, and they all reported significant subjective benefit from their HAs, which were fitted to the prescriptive target recommended by the device specific fitting software. The HA fitting was confirmed with real-ear measurements as required. However, no free field thresholds were measured for the HAs in order to quantify the bimodal benefit. Therefore, if assessed for the best aided condition, some of our bimodal users might have been categorized as unilateral users, which could affect the comparative analysis between listening modalities.

## 6. Conclusions

Our data confirm that it is possible to create a comprehensive SIN test battery that includes different types of SIN tests, and still produces comparable results in a diverse patient population. The data reported here provide references for the FMST, FINSIMAT and FDTT in CI users, and are the first data for simplified matrix tests in adult CI users. Similar CI user reference data for other SIN tests would provide valuable information for clinicians assessing the rehabilitation results or CI candidacy, and for the scientific community at large in comparing results from different centers and studies.

## Figures and Tables

**Table 1:** The characteristics of the speech intelligibility in noise tests used in the study.

	Finnish Matrix Sentence Test (FMST)	Finnish Simplified Matrix Sentence Test (FINSIMAT)	Finnish Digit Triplet Test (FDTT)
**Test material**	Five-word sentences	Three-word pseudo-sentences	Digit triplets (*two-six-nine*)
**Content of a test list**	20 sentences	14 pseudo-sentences	30 triplets
**Step size for the adaptive procedure**	Decreases progressively	Decreases progressively	Fixed at 2 dB SNR
**Scoring method**	Word scoring	Word scoring	Triplet scoring
**Method for determining the SRT**	Maximum likelihood method	Maximum likelihood method	The mean SNR of the last 27 triplets
**Reference value (SD) for normal-hearing listeners ^1^**	−9.7 (0.7) dB SNR	−11.2 (1.0) dB SNR	−10.8 (0.5) dB SNR

^1^ Reference values were obtained monaurally with headphones: Finnish matrix sentence test [26], Finnish simplified matrix sentence test [40], and Finnish digit triplet test [41]; SNR: signal-to-noise ratio.

**Table 2:** Summary of the demographics and test results of the participants.

	Age ≤65	Age >65	All
**Number of participants**	55	25	80
Kuopio	33 (61%)	21 (39%)	54
Helsinki	22 (85%)	4 (15%)	26
**Age**, median (range), y	53 (17–65)	73 (66–85)	60 (17–85)
**Length of CI experience,** median (range), mo	32 (5–229)	14 (6–87)	26 (5–229)
**Device configuration**			
CI	29 (66%)	15 (34%)	44
CI + HA	8 (57%)	6 (43%)	14
CI + CI	18 (82%)	4 (18%)	22
**Cochlear implant manufacturer**			
Advanced Bionics	5 (45%)	6 (55%)	11
Cochlear	20 (67%)	10 (33%)	30
Med-El	29 (74%)	10 (26%)	39
**Speech perception scores**			
**FMST, +10 dB SNR** median (range), %	98 (78–100)	96 (76–100)	97 (76–100)
**FMST, adaptive procedure** mean (SD), dB SNR	−4.2 (2.0)	−4.0 (2.1)	−4.2 (2.1)
**FINSIMAT, adaptive procedure** mean (SD), dB SNR	−5.0 (2.0)	−5.1 (1.8)	−5.0 (1.9)
**FDTT, adaptive procedure** mean (SD), dB SNR	−4.7 (2.0)	−4.4 (1.6)	−4.6 (1.9)

FMST, Finnish matrix sentence test; FINSIMAT, Finnish simplified matrix sentence test; FDTT, Finnish digit triplet test; SNR, signal-to-noise ratio.

## Data Availability

The data presented in this study are available on request from the corresponding author.
